# Prevalence of Psychosomatic and Genitourinary Syndrome Among Menopausal Women: A Systematic Review and Meta-Analysis

**DOI:** 10.3389/fmed.2022.848202

**Published:** 2022-03-03

**Authors:** Nik Hussain Nik Hazlina, Mohd Noor Norhayati, Ismail Shaiful Bahari, Nik Ahmad Nik Muhammad Arif

**Affiliations:** ^1^Women's Health Development Unit, School of Medical Sciences, Universiti Sains Malaysia, Health Campus, Kubang Kerian, Malaysia; ^2^Department of Family Medicine, School of Medical Sciences, Universiti Sains Malaysia, Health Campus, Kubang Kerian, Malaysia

**Keywords:** menopause, prevalence, psychosomatic, genitourinary syndrome, symptom

## Abstract

**Introduction:**

The menopausal transition represents the passage from reproductive to non-reproductive life and is characterized by a number of menstrual disturbances. We systematically reviewed the evidence on the prevalence of psychosomatic and genitourinary syndrome among menopausal women and compared the risk of symptoms between premenopausal, perimenopausal, and post-menopausal women.

**Methods:**

We performed a systematic search in MEDLINE, CINAHL, and ScienceDirect through March 2021. Case series/reports, conference papers and proceedings, articles available only in abstract form, editorial reviews, letters of communication, commentaries, systematic reviews, and qualitative studies were excluded. Two reviewers independently extracted and assessed the quality of data using the Joanna Briggs Institute Meta-Analysis. The outcomes were assessed with random-effects model using the Review Manager software.

**Results:**

In total, 29 studies had a low risk of bias and were included in the review. Our findings showed that the pooled prevalence of somatic symptoms in post-menopausal women (52.6%) was higher than in the premenopausal and perimenopausal stages (34.6 and 39.5%, respectively). There was a low prevalence of psychological symptoms in premenopausal women (28.4%). The genitourinary syndrome was highest among post-menopausal women (55.1%), followed by perimenopausal (31.9%) and premenopausal (19.2%) women.

**Conclusion:**

Post-menopausal women have a higher risk of experiencing menopausal symptoms particularly genitourinary syndrome than premenopausal and perimenopausal women. It is pertinent for healthcare professionals to evaluate the symptoms in order to provide them with a better quality of life.

**Systematic Review Registration:**

https://www.crd.york.ac.uk/prospero/display_record.php?ID=CRD42021235958

## Introduction

Natural menopause occurs at a median age of 51 years in Western societies and is assumed to be caused by ovarian follicle exhaustion (1). Based on the Stages of Reproductive Aging Workshop (STRAW) criteria, menopause is defined as 12 or more months of amenorrhea following the final menstrual period (2). Perimenopause is defined as the onset of a varied cycle duration (early stage), two or more missing periods, and at least 2 months of amenorrhea (late-stage) (2). As the population ages, a growing proportion of women are living decades after menopause. At menopause, about 80% of women have vasomotor symptoms (VMS), such as hot flushes and night sweats, and about 20% find them bothersome enough to seek therapy (3).

Menopause is defined as the permanent cessation of menstruation resulting from the loss of ovarian follicular activity. Natural menopause is recognized to have occurred after 12 consecutive months of amenorrhea, for which there is no other obvious pathological or physiological cause. Menopause occurs with the final menstrual period which is known with certainty only in retrospect a year or more after the event. Perimenopause should include the time immediately prior to the menopause (when the endocrinological, biological and clinical features of approaching menopause commence) and the first year after menopause. The term premenopause is often used ambiguously, either to refer to the 1 or 2 years immediately before the menopause or to refer to the whole of the reproductive period prior to the menopause. The group recommended that the term be used consistently in the latter sense to encompass the entire reproductive period up to the final menstrual period. Post-menopause is defined as dating from the final menstrual period, regardless of whether the menopause was induced or spontaneous (4).

Methods for determining when perimenopause begins (5), and a distinct set of STRAW criteria was developed (2). A variety of menstruation disturbances characterize the menopausal transition. The VMS is a common occurrence, sometimes accompanied by psychosomatic symptoms such as mood swings, anxiety, stress, and genitourinary syndrome of menopause. This includes genital symptoms such as dryness, burning, and irritation, as well as sexual symptoms such as lack of lubrication, discomfort or pain, poor function, and urinary symptoms such as urgency, dysuria, and recurrent urinary tract infections (6). All these symptoms have a significantly negative impact on the quality of life of many women (7). The menopausal transition can be a difficult time in a woman's life when it comes to healthy aging. These distressing symptoms do not affect all middle-aged women. During the menopausal transition, a third of women do not report any problems. Another third has mild to moderate symptoms, and the remaining third has moderate to severe symptoms that necessitate therapy (8).

This systematic review and meta-analysis aimed to determine the prevalence of psychosomatic and genitourinary syndrome among menopausal women. It helps to understand the symptoms better and serves as a basis for more appropriate countermeasures to this natural phenomenon. This updated review could be used in primary preventative care to help patients control and prevent diseases that harm their quality of life and mental health.

## Materials and Methods

A systematic review and meta-analysis were conducted to assess the prevalence of psychosomatic and genitourinary syndrome among menopausal women. The study followed the Preferred Reporting Items for Systematic Reviews and Meta-Analyses (PRISMA) guidelines (9) and registered in the PROSPERO (CRD42021235958).

### Search Strategy and Eligibility Criteria

We (N.A.N.M.A., N.H.N.H.) performed a systematic search in MEDLINE (PubMed), CINAHL (EBSCOhost), and ScienceDirect using the search terms “psycholog^*^,” “mental,” “anxiety,” “depression,” “stress,” and “menopaus^*^.” These terms were adaptable to the different electronic databases. To determine their eligibility for inclusion in this study, all studies published from the beginning of the databases until March 2021 were retrieved. The search was limited to full-text articles written in English. The reference lists of the listed citations were cross-checked to locate other potentially eligible research.

The inclusion criteria were studies that reported the prevalence of psychosomatic and genitourinary syndrome among menopausal women. In this review, the definitions of premenopausal, perimenopausal and post-menopausal were as defined by the researchers in their studies. Studies with cross-sectional, case-control, and cohort designs published in English and conducted in the community or at a health institution level were included. Case series/reports, conference papers and proceedings, articles available only in abstract form, letters of communication, editorial reviews, systemic reviews, commentaries, and qualitative studies were excluded.

### Study Selection and Quality Assessment

**Figure 1:**
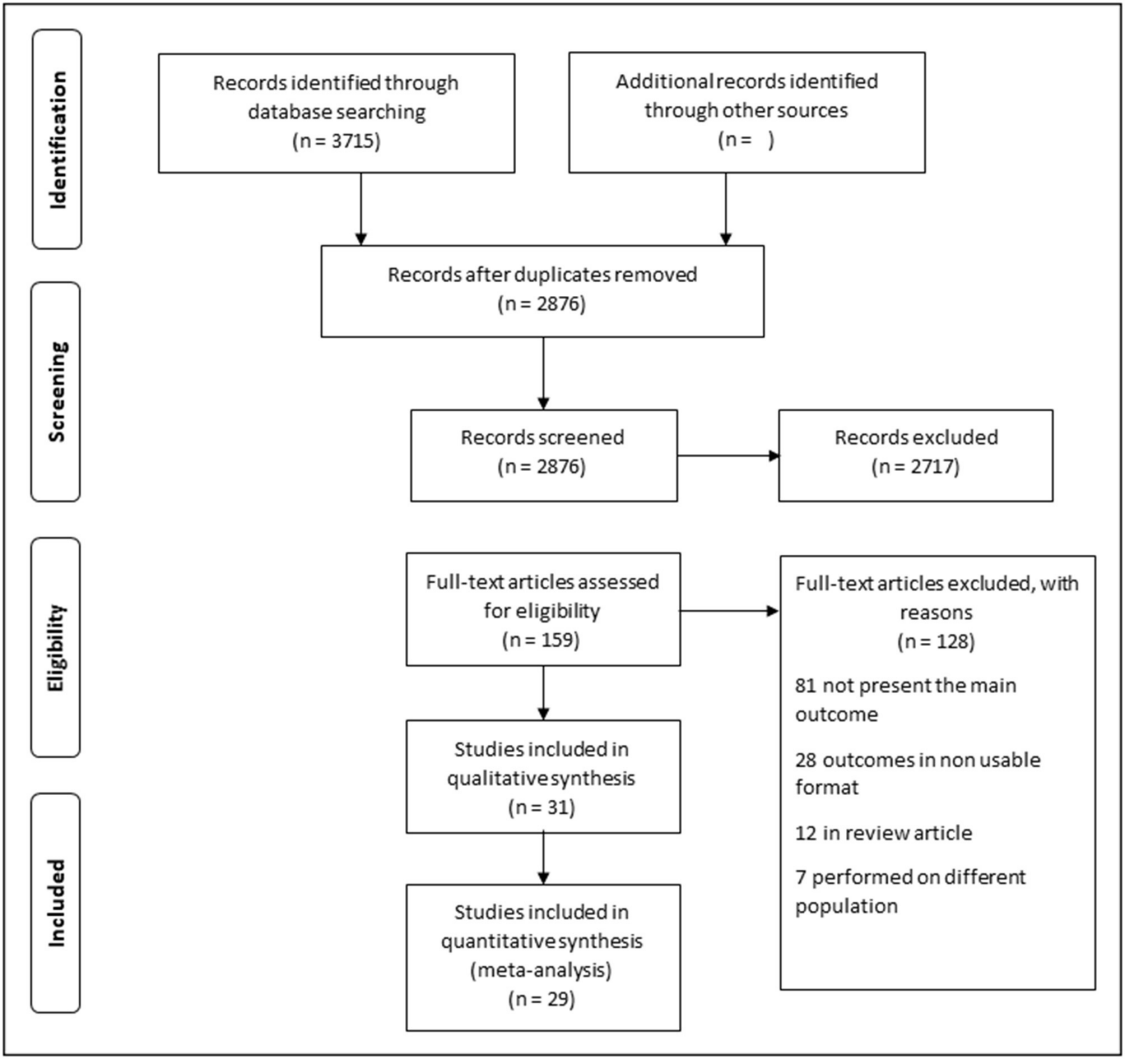
Flowchart showing the studies on psychosomatic and genitourinary syndrome among menopausal women.

All the records identified using our search strategy were exported to EndNote X8 software (Clarivate Analytics, Philadelphia, PA). Duplicate articles were removed. To determine their appropriateness, the entire texts of the relevant research were obtained and thoroughly examined. A consensus discussion was held between the two reviewers (N.A.N.M.A., N.H.N.H.) in the event of a conflict, and a third reviewer (M.N.N.) was consulted. The search method is presented in the PRISMA flowchart, which shows the studies that were included and excluded with reasons for their exclusion (Figure 1).

To examine the data quality, a critical assessment was carried out using the Joanna Briggs Institute Critical Appraisal Checklist for cross-sectional, case-control, and cohort studies (10). Two reviewers (N.A.N.M.A., N.H.N.H.) performed independent bias evaluations. When more than 70% of the answers were “yes,” the risk of bias was considered low, moderate when 50–69% of the answers were “yes,” and high when up to 49% of the answers were “yes.” This review omitted studies that had a moderate or high risk of bias.

### Data Extraction Process and Statistical Analysis

Two reviewers (N.A.N.M.A., N.H.N.H.) independently extracted the data using Microsoft Excel (Microsoft Corporation, Redmond, WA). The process included the first author, publication year, study design and setting, study location, study population, sample size, psychological impact, menopausal definition, and data in calculating the effect estimates for psychosomatic and genitourinary symptoms. In the event that disagreement occurred, it was dissolved by discussion between the two review authors or by the third author (M.N.N.).

The results were presented as odds ratios (OR) with 95% confidence intervals (CI). The analysis was performed using Review Manager software version 5.4 (Nordic Cochrane Centre, Copenhagen, Denmark). A random-effects model was employed to pool the data. The *I*^2^ statistic was used to assess heterogeneity using the following guidelines: 0–40% may not be significant, 30–60% may represent moderate heterogeneity, 50–90% may represent substantial heterogeneity, and 75–100% may indicate significant heterogeneity (11). A subgroup analysis was performed on the basis of the study location (developed and developing countries) and comorbidities (presence or absence thereof) if an adequate number of studies were available. If publication bias was suspected, funnel plots and Egger's test were performed, with a *p*-value of <0.05 indicating statistical significance.

## Results

### Study Characteristics

Through the electronic search, a total of 3,715 articles were identified, and 2,876 articles were found to be eligible after duplicate records were removed. A further 2,717 articles were excluded based on their titles or abstracts. The full texts of 159 articles were searched. Subsequently, 128 articles were excluded: 40 did not present the main outcomes, 41 had no outcomes of interest, 28 studies reported the outcomes in an unusable format, 12 were review articles, and seven were performed among different populations. Thirty-one studies underwent quality assessments, of which two had a moderate or high risk of bias and were excluded. The remaining 29 articles had a low risk of bias. Of these, 28 were cross-sectional studies, and one was a cohort. A quantitative assessment was then performed (Figure 1).

The women in the included studies were generally recruited from the general population or those who attended health clinics or gynecology clinics before starting treatment of menopausal complaints. They were not on treatment for menopausal symptoms except for a few studies that had <10% of the samples on hormone replacement therapy (12–16). One study reported that in all menopausal groups, women who used HRT did not differ from non-users in having at least one bothersome symptom (17).

Different countries were included in this review. Four studies were conducted in Spain (15, 16, 18, 19), two each in Ecuador (13, 20), Turkey (21, 22), Korea (23, 24), and China (25, 26), and one each in India (27), Bangladesh (28), Saudi Arabia (29), the United States (30), Oman (31), Africa (32), Finland (17), Israel (33), Japan (34), Russia (35), Chile (36), Brazil (37), Poland (38), Italy (39), Nepal (40), Singapore (14), and Jordan (12). The smallest sample size was 187 (27) and the largest 2,886 (34). Overall, this study included 21,375 women (Table 1).

**Table 1:** Summary of the research articles included in the systematic review and meta-analysis on menopause (*n* = 29).

**No**	**References**	**Country**	**Study design**	**Sample size (*n*)**	**Perimenopause (*n*)**	**Quality assessment (%)**
1	Ahsan et al. (27)	India	Cross- sectional	187	92	87.5
2	Alam et al. (28)	Bangladesh	Cross- sectional	326	79	100
3	AlJoharah et al. (29)	Saudi Arabia	Cross-sectional	490	75	100
4	Arakane et al. (18)	Spain	Cross- sectional	340	72	100
5	Avis et al. (30)	United State	Cross-sectional	881	539	87.5
6	Chedraui et al. (13)	Ecuador	Cross-sectional	300	144	87.5
7	Chedraui et al. (20)	Ecuador	Cross-sectional	404	127	100
8	El Shafie et al. (31)	Oman	Cross-sectional	472	73	100
9	Engida et al. (32)	Africa	Cross-sectional	226	151	100
10	Moilanen et al. (17)	Finland	Cross-sectional	1,427	184	100
11	Becker et al. (33)	Israel	Cross-sectional	189	40	100
12	Kuninori et al. (34)	Japan	Cross-sectional	2,886	211	100
13	Chernicenko et al. (35)	Russia	Cross-sectional	416	416	100
14	Binfa et al. (36)	Chile	Cross-sectional	300	46	100
15	Coronado et al. (19)	Spain	Cross-sectional	308	69	87.5
16	Gozuyesil et al. (22)	Turkey	Cross-sectional	317	21	100
17	Ornat et al. (16)	Spain	Cross-sectional	260	61	87.5
18	Cuadros et al. (15)	Spain	Cross-sectional	235	6	100
19	da Silva et al. (37)	Brazil	Cross-sectional	1,415	143	100
20	Raczkiewicz et al. (38)	Poland	Cross-sectional	300	143	100
21	Shin et al. (24)	Korea	Cross-sectional	2,400	402	100
22	Sun et al. (26)	China	Cross-sectional	2,046	141	100
23	Fabbrini et al. (39)	Italy	Cross-sectional	334	42	87.5
24	Koirala et al. (40)	Nepal	Cross-sectional	240	86	87.5
25	Ryu et al. (23)	Korea	Cross-sectional	2,481		87.5
26	Chim et al. (14)	Singapore	Cross-sectional	495	124	87.5
27	Biri et al. (21)	Turkey	Cross-sectional	1,049		87.5
28	Abdelrahman et al. (12)	Jordan	Cross-sectional	193	35	100
29	Luo et al. (25)	China	Cohort	458	78	72.7

### Prevalence of Premenopausal, Perimenopausal, and Post-menopausal Women

**Figure 2:**
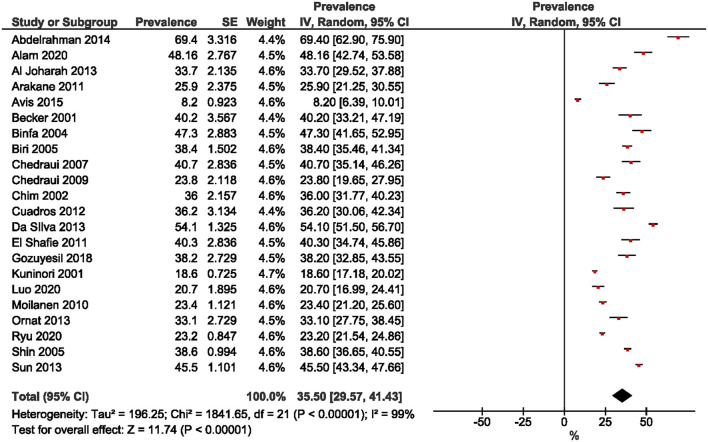
Forest plot of the prevalence of premenopausal women.

**Figure 3:**
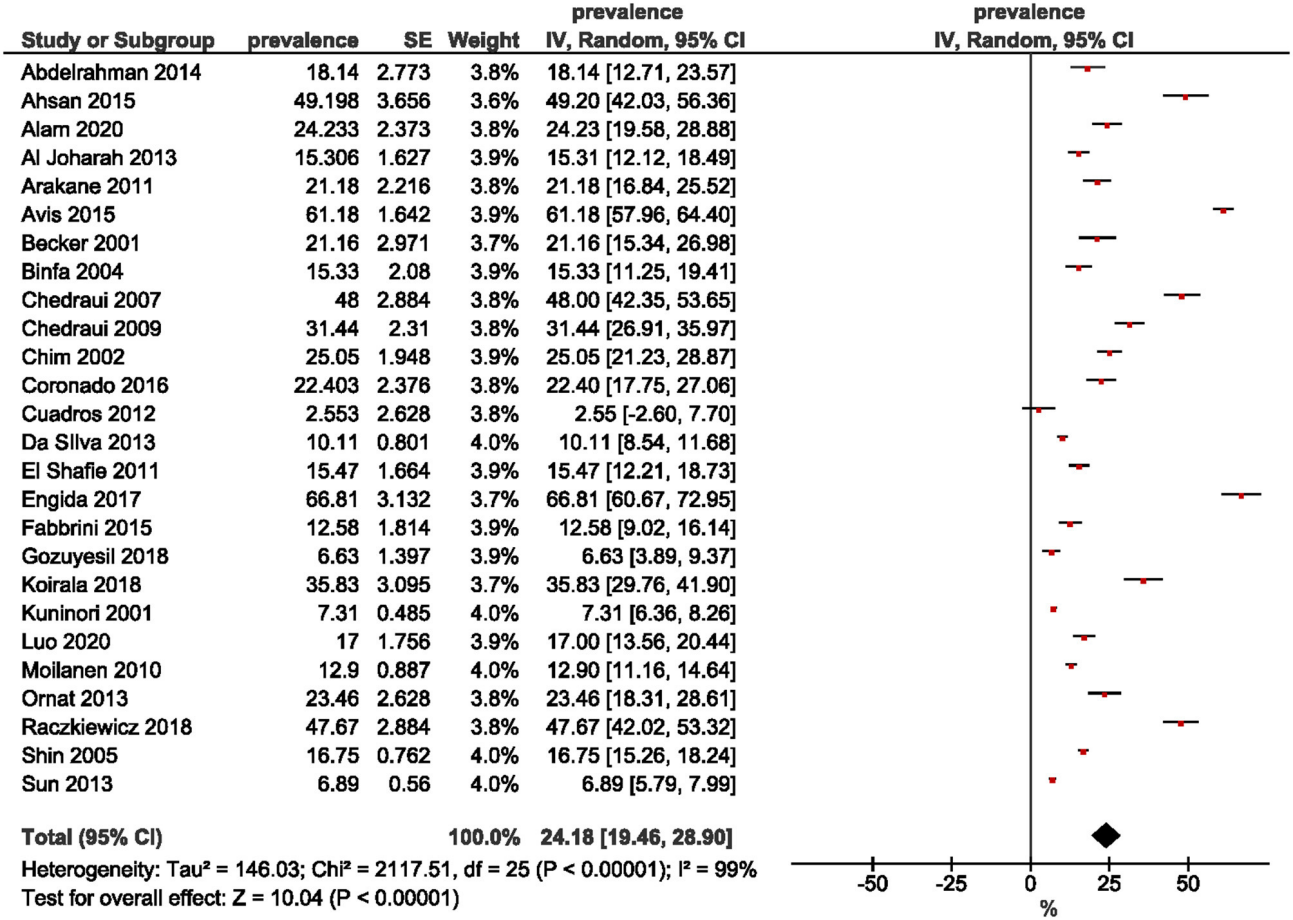
Forest plot of the prevalence of perimenopausal women.

**Figure 4:**
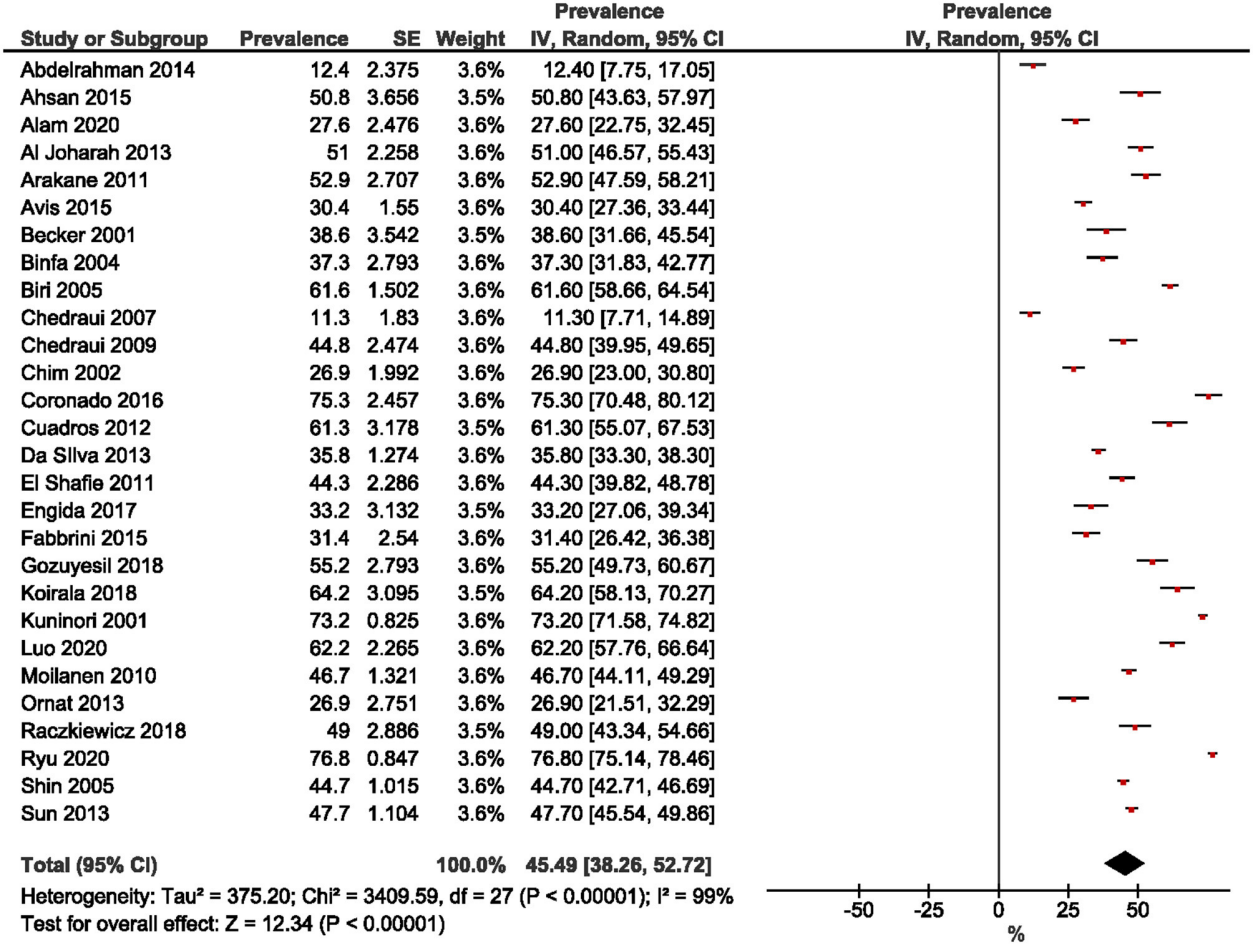
Forest plot of the prevalence of post-menopausal women.

Twenty-two studies were included in the estimation of the prevalence of premenopausal women. A wide range was observed, from 8.2% (30) to 69.4% (12). The overall pooled prevalence of premenopausal women was 35.5% (95% CI: 29.57, 41.43; Figure 2). The prevalence of perimenopausal women was estimated from 26 studies and ranged from 2.55% (15) to 66.81% (32). The pooled prevalence was 24.18% (95% CI: 19.46, 28.90; Figure 3). The prevalence of post-menopausal women was derived from 28 studies and ranged from 11.3% (13) to 76.80% (23). The pooled prevalence was 45.49% (95% CI: 38.26, 52.72; Figure 4). Egger's test showed significant publication bias for premenopausal (*P* = 0.026), perimenopausal (*P* < 0.001), and postmenopausal (*P* = 0.014) women.

### Prevalence of Symptoms Among Menopausal Women

The Menopause Rating Scale was used to determine the prevalence of menopausal symptoms, which comprises 11 items divided into three subscales: (a) somatic symptoms: hot flashes, cardiac symptoms, sleep disturbances, and joint and muscle discomfort; (b) psychological symptoms: depressive mood, irritability, anxiety, and physical and mental exhaustion; and (c) genitourinary syndrome: sexual problems, bladder problems, and vaginal dryness.

Table 2 shows the percentage of women who experienced symptoms according to the menopausal stage. Somatic symptoms in post-menopausal women were higher (52.6%) compared to the premenopausal and perimenopausal stages (34.6 and 39.5%, respectively). The prevalence of psychological symptoms in premenopausal women was low (28.4%) but nearly the same among post-menopausal (49.4%) and perimenopausal (42.1%) women. The prevalence of genitourinary syndrome was different in all three groups: the highest prevalence was among post-menopausal women (55.1%), followed by perimenopausal (31.9%) and premenopausal (19.2%) women.

**Table 2:** The prevalence of symptoms among menopausal women according to menopausal stage.

**Symptom**	**Prevalence (%)**		
	**Premenopausal**	**Perimenopausal**	**Post-menopausal**
**(a) Somatic symptom**	**34.6**	**39.5**	**52.6**
Hot flashes	22.2 (13, 17, 31)	52.9 (13,17,27,31,32)	59.7 (13, 17,27,31, 2)
Cardiac symptom	11.0 (13,17,31)	26.4 (13,17 27,31,32)	41.8 (13,17,27,31, 32)
Sleep disturbance	24.9 (13,17,18,24,26,31)	36.4 (13,17,24,26,27,29,31,32,39)	45.1 (13,17,18,24,26,27,31,38)
Joint and muscle discomfort	53.8 (13,21,31)	46.4 (13,31,32)	69.9 (13,21,31,32)
**(b) Psychological symptom**	**28.4**	**42.1**	**49.4**
Depressive mood	32.4 (13,17,21,31)	51.9 (13,17,27,31,32)	43.7 (13,17,27,31,32)
Anxiety	35.8 (13,21,31)	54.6 (13,27,31,32)	52.0 (13,21,27,31,32)
Irritability	23.6 (13,17,21,31)	27 (13,17,31,32)	51.7 (13,17,31,32)
Physical and mental exhaustion	21.5 (13,17,31)	31.9 (13,17,31,32)	52.2 (13,17,31,32)
**(c) Genitourinary syndrome of menopuse**	**19.2**	**31.9**	**55.1**
Sexual problem	22.4 (13,31)	45 (13,32,32)	56.1 (13,31,32)
Bladder problem	33.6 (13,31)	31.9 (13,27,31,32)	62.1 (13,27,31,32)
Vaginal dryness	3.9 (13,31)	31.9 (13,31,32)	39.9 (31,32)

In terms of somatic symptoms, post-menopausal women had the highest prevalence (52.6%) of hot flashes, which were present in 34.6% of premenopausal and 39.5% of perimenopausal women. The prevalence of cardiac symptoms and sleep disturbances was <50% across all three menopausal phases. Meanwhile, for joint and muscle discomfort, post-menopausal women had the highest prevalence (69.9%).

Among the psychological symptoms, perimenopausal women had the highest prevalence of depressive mood (51.9%) and anxiety (54.6%), followed by post-menopausal and then premenopausal women. The prevalence of irritability and physical and mental exhaustion was highest among post-menopausal women at 51.7 and 52.2%, respectively. Post-menopausal women had the highest prevalence of genitourinary syndrome: 56.1% had sexual problems, 62.1% had bladder problems, and 39.9% had vaginal dryness.

### Comparison of Symptoms Between Premenopausal and Perimenopausal Women

Symptoms between premenopausal and perimenopausal women were compared using the Menopause Rating Scale. For somatic symptoms, there were significant differences in the occurrence of hot flashes (OR: 4.16; 95% CI: 2.15, 8.02), cardiac symptoms (OR: 1.69; 95% CI: 1.04, 2.76), and sleep disturbances (OR: 1.51; 95% CI: 1.13, 2.01) between the groups, but no difference for joint and muscle discomfort (OR: 1.47; 95% CI: 0.04, 58.31). There were no significant differences in psychological symptoms aside from physical and mental exhaustion, where the prevalence was higher among perimenopausal than premenopausal women (OR: 2.19; 95% CI: 1.33, 3.58). For genitourinary syndrome, a significant difference was only apparent for sexual problems (OR: 9.62; 95% CI: 5.87, 15.76).

### Comparison of Symptoms Between Premenopausal and Post-menopausal Women

A comparison of somatic symptoms between premenopausal and post-menopausal women showed a significant difference for all four symptoms. The odds of experiencing hot flashes were 4.72 (95% CI: 1.99, 11.21) times higher among post-menopausal than premenopausal women. The findings were similar for cardiac symptoms (OR: 4.66; 95% CI: 1.28, 16.92), sleep disturbances (OR: 2.24; 95% CI: 1.84, 2.73), and joint and muscle discomfort (OR: 2.08; 95% CI: 1.68, 2.57). In terms of psychological symptoms, anxiety was the only symptom showing no significant difference between the women (OR: 1.18; 95% CI: 0.72, 1.92). There was a significant difference for the rest of the symptoms; namely, depression (OR: 1.39; 95% CI: 1.09, 1.76), irritability (OR: 2.18; 95% CI: 1.12, 4.23), and physical and mental exhaustion (OR: 4.07; 95% CI: 1.45, 11.44), while for the genitourinary syndrome, there was a significant difference for sexual (OR: 9.01; 95% CI: 4.68, 17.36) and bladder problems (OR: 2.72; 95% CI: 1.75, 4.23).

### Comparison of Symptoms Between Perimenopausal and Post-menopausal Women

In the comparison of symptoms between perimenopausal and post-menopausal women, sleep disturbances were the only somatic symptom showing a significant difference (OR: 0.7; 95% CI: 0.53, 0.92) in favor of perimenopausal women. Among the psychological symptoms, physical and mental exhaustion was the only symptom for which there was a significant difference (OR: 0.31; 95% CI: 0.12, 0.83). Lastly, in terms of the genitourinary syndrome, there was only a significant difference for bladder problems (OR: 0.23; 95% CI: 0.09, 0.62).

## Discussion

This study aimed to determine the pooled prevalence of psychosomatic and genitourinary syndrome among menopausal women. In this meta-analysis, the pooled prevalence of premenopausal, perimenopausal, and post-menopausal women was 35.5, 24.18, and 45.49%, respectively. The wide range of prevalence for each of the three menopausal stages was due to the different study settings, where 21 (12, 13, 15, 16, 18–24, 27, 29–31, 36–40) studies were conducted in medical centers, and eight (14, 17, 25, 28, 32–35) were in the community. Methodological techniques such as sampling methods and tools, together with study implementation may also account for differences in estimated prevalence among studies.

In subscale of somatic symptoms, the prevalence of hot flashes is 24–93% among post-menopausal women, with the symptoms varying from mild to severe in the general population (41). The first 2 years after menopause are when hot flashes are most common, but they can last for years: 64% of women have reported symptoms lasting for 5 years, 26% for 10 years, and 10% for more than 10 years (42). Comparison between perimenopausal and post-menopausal women had nearly the same prevalence (52.9 and 59.7%, respectively). Notably, the odds of having hot flashes during perimenopause and post-menopause increased about four times compared to during premenopause. Menopausal symptoms emerge early in the premenopausal stage, even before monthly irregularities appear (43). Menopausal symptoms were seen in young premenopausal women (aged 40–44 years) with normal menstrual cycles, but their frequency increased with age and menopausal phase (43). The symptoms lasted for years and continued until five or more years post menopause with the same frequency and intensity as during the menopausal transition (44, 45).

In this study, post-menopausal women had the highest prevalence of cardiac symptoms at 52.6%, reflecting their risk of having cardiovascular disease. A meta-analysis on the development of cardiovascular disease among menopausal women reported that vasomotor and other menopausal symptoms were associated with an increased risk of chronic heart disease, stroke, and cardiovascular disease (46). Meanwhile, the prevalence of sleep disturbances and joint and muscle discomfort among premenopausal women was 24.9 and 53.8%, respectively, which was similar to the results of a systematic review of middle-aged Asian women, where the prevalence of sleep disturbances was 6.1–54% and joint and muscle discomfort 10.3–82.1% (47).

In the sleep disturbances subgroup, all the findings were significant, with post-menopausal women at higher risk of sleep disturbances than premenopausal (OR: 2.24) and perimenopausal (OR: 0.70) women. Similar findings were published in a meta-analysis in 2014, where the odds for post-menopausal women were 1.67 times higher than for premenopausal women and 1.09 times higher than for perimenopausal women (48). This significant association is also supported by other reviews (49, 50). Increased sleep disturbances during the menopausal transition are likely due to hot flashes occurring during the night because women with hot flashes may also experience nocturnal awakening unrelated to VMS (49). Around 60–80% of women experience hot flashes during the menopausal transition (51). They persist for 4–5 years (52) and commonly lead to sleep disturbances among perimenopausal and post-menopausal women.

Menopause can be psychologically distressing for women. Subscale of psychological symptoms showed that the occurrence of clinical depression in this study did not increase significantly among premenopausal and post-menopausal women, with perimenopausal women as the reference group. However, depression was 1.39 times more likely for post-menopausal than premenopausal women. A meta-analysis found similar results: post-menopausal women were 1.5 times more likely to experience depression than premenopausal women (53). The study also reported that post-menopausal women were 4.3 times more predisposed to major depression than premenopausal women (53). Depressive symptoms are more common during the menopausal transition for a variety of reasons, with earlier depression being one of them (54).

In our study, perimenopausal women had the highest prevalence of anxiety, which is consistent with the theory that anxiety levels rise throughout menopause and diminish following menopause (55, 56). We found that irritability was highest among post-menopausal women, which is not consistent with other studies (57–60). Women in the early stages of menopause show significantly higher levels of irritability than premenopausal and post-menopausal women. Moreover, we found a higher risk of exhaustion among post-menopausal women than the other groups: perimenopausal women had a 1.78 times higher risk of developing psychological symptoms than premenopausal women and a 1.83 higher risk than premenopausal women.

Concerning genitourinary syndrome, the prevalence of sexual problems and vaginal dryness in post-menopausal women was 56.1 and 39.9%, respectively. This is similar to a large cohort study (61) among a Western population, where the estimated prevalence of vulvovaginal symptoms was found to be 45–63%. A study in Korea (62) found that 49% of post-menopausal women experienced the same symptoms. A study in Turkey revealed that low sexual function was higher among post-menopausal than premenopausal women (63). Reports have shown that 40% of women who experience vulvovaginal symptoms have sexual dysfunction, 24% lack sexual desire, 34% have arousal difficulties, and 19% experience difficulties with orgasm (64).

## Strengths and Limitations

This review demonstrated the prevalence for each menopausal phase and the associated symptoms across different countries. By expressing the symptoms quantitatively, they could be compared, thus providing a comprehensive overview of the various studies' results. In general, a meta-analysis of prevalence study can provide useful information about disease burden, such as finding discrepancies between populations and regions (65). However, this study was not without limitations. Diverse instruments were used to determine the symptoms during the menopausal transition, which reduced the comparability of the results. This review only included English-language publications; however, there is no evidence of a systematic bias in the use of language restrictions in systematic review-based meta-analyses in conventional medicine (66). We attempted to produce convincing evidence by including high quality studies. Two moderate and low quality studies were excluded from the review. However, the exclusion itself may possibly introduce potential biases in the review process.

## Conclusions

This review demonstrated that post-menopausal women are at a higher risk of menopausal symptoms than premenopausal and perimenopausal women. Additionally, post-menopausal women have a significantly higher risk of experiencing genitourinary syndrome than other groups of symptoms compared to premenopausal and perimenopausal women. Healthcare practitioners must regularly evaluate the symptoms of postmenopausal women in order to offer them a higher quality of life. Future research should focus on various phases of menopausal symptoms in order to acquire a comprehensive picture of menopause.

## Search Strategy

1. psychology
2. mental
3. anxiety
4. depression
5. stress
6. #1 OR #2 OR #3 OR #4 OR #5
7. menopause
8. #6 AND #7

## Data Availability Statement

The original contributions presented in the study are included in the article/supplementary material, further inquiries can be directed to the corresponding author/s.

## Author Contributions

NHNH, MNN, and ISB: conceptualization and methodology. NHNH and MNN: validation, resources, and visualization. NHNH, MNN, ISB, and NANMA: formal analysis, investigation, and writing—review and editing. NHNH, MNN, and NANMA: data curation. NANMA: writing—original draft preparation. NHNH: supervision. MNN: project administration. All authors have read and agreed to the published version of the manuscript.

## Conflict of Interest

The authors declare that the research was conducted in the absence of any commercial or financial relationships that could be construed as a potential conflict of interest.

## Publisher's Note

All claims expressed in this article are solely those of the authors and do not necessarily represent those of their affiliated organizations, or those of the publisher, the editors and the reviewers. Any product that may be evaluated in this article, or claim that may be made by its manufacturer, is not guaranteed or endorsed by the publisher.
